# Reliability of recommendations to reduce a fracture of the distal radius

**DOI:** 10.1080/17453674.2020.1846853

**Published:** 2020-11-13

**Authors:** Emily Z Boersma, Joost T P Kortlever, Maria W G Nijhuis-Van Der Sanden, Michael J R Edwards, David Ring, Teun Teunis

**Affiliations:** a Radboud University Medical Center, Radboud Institute for Health Sciences, Department of Surgery, Nijmegen, the Netherlands;; b Department of Surgery and Perioperative Care, Dell Medical School, The University of Texas at Austin, USA;; c Radboud University Medical Center, Radboud Institute for Health Sciences, Department of IQ Healthcare, Nijmegen, the Netherlands;; d Department of Plastic Surgery, University Medical Center Utrecht, Utrecht, The Netherlands

## Abstract

Background and purpose — It is unclear what degree of malalignment of a fracture of the distal radius benefits from reduction. This study addressed the following questions: (1) What is the interobserver reliability of surgeons concerning the recommendation for a reduction for dorsally displaced distal radius fractures? (2) Do expert-based criteria for reduction improve reliability or not?

Methods — We sent out 2 surveys to a group of international hand and fracture surgeons. On the first survey, 80 surgeons viewed radiographs of 95 dorsally displaced (0° to 25°) fractures of the distal radius. The second survey randomized 68 participants to either receive or not receive expert-based criteria for when to reduce a fracture and then viewed 20 radiographs of fractures with dorsal angulation between 5° and 15°. All participants needed to indicate whether they would advise a reduction or not.

Results — In the 1st study, the interrater reliability of advising a reduction was fair (kappa 0.31). Multivariable linear regression analyses indicated that each additional degree of dorsal angulation increased the chance of recommending a reduction by 3%. In the 2nd study, reading criteria for reduction did not increase interobserver reliability for recommending a reduction.

Interpretation — There is notable variation in recommendations for reduction that is not accounted for by surgeon or patient factors and is not diminished by exposure to expert criteria. Surgeons should be aware of their biases and develop strategies to inform patients and share the decision regarding whether to reduce a fracture of the distal radius.

Many aspects of distal radius fracture (DRF) management are debated (Koval et al. 2014, Mauck and Swigler 2018). For example, it is unclear what degree of malalignment of a DRF benefits from reduction (Mackenney et al. 2006, Dario et al. 2014). Some guidelines address criteria for the adequacy of a reduction, but there is less written about recommendations for when to reduce a fracture. The Clinical Practice Guideline from the American Academy of Orthopedic Surgeons and the Dutch guideline, for example, do not address when to reduce a fracture (AAOS 2009, Brink et al. 2010).

The international distal radius fracture study group suggested the following criteria for offering a reduction: dorsal tilt of the articular surface on the lateral radiograph more than 10°, intra-articular displacement more than 2 mm, ulnar positive variance of more than 5 mm, and ulnar-ward inclination of the articular surface on the posteroanterior radiograph less than 15° (Nelson 2006). However, these recommendations are based on little data.

In a survey study in the Netherlands, there was limited inter-surgeon agreement on recommendations for treatment after reduction of a fracture of the distal radius after introducing a national guideline (Pijls et al. 2016). Understanding the sources of practice variation is the first step towards reducing unhelpful and unwarranted variation. Unwarranted practice variation indicates the need to optimize the balance of benefits and harms, while limiting unhelpful use of resources (Birkmeyer et al. 2013, Saving et al. 2018).

In order to learn from variation, we first need to know what drives it. This study addresses the interobserver reliability of surgeons recommending a reduction of a DRF and whether reading expert-based criteria before advising a reduction has an effect on the reliability of advising a reduction. The following questions were addressed: (1) what is the interobserver reliability of the recommendation to reduce a DRF? (2) Is there a difference in interobserver reliability based on surgeon characteristics, fracture types, and patient characteristics? (3) What radiographic factors and patient characteristics are independently associated with recommending a reduction? And finally (4), do expert-based criteria increase interobserver reliability?

## Methods

### Study design

The 1st survey addressed recommendation for reducing a DRF. The 2nd tested the influence of expert-based criteria for recommending a reduction for a DRF. Surveys were created and distributed through SurveyMonkey (Palo Alto, CA, USA).

### Participants

Members (surgeons) of the Science of Variation Group (SOVG) were invited to participate in the 1st study and 2 months later they were then invited to participate in the 2nd study. The SOVG is an international collaboration of orthopedic surgeons, plastic surgeons, and fracture surgeons that studies variation in the definition, interpretation, classification, and treatment of human illness (https://sites.google.com/site/scienceofvariationgroup/home). Only a subset participates regularly in the surveys, and even regular participants respond to surveys only in their region of expertise, so it is not possible to measure a meaningful response rate. For these studies we invited surgeons who are specialized in upper extremity and hand surgery.

### Recommendations for reduction

For the survey regarding recommendations for reduction, we selected 95 consecutive radiographs from patients with a DRF treated in the Radboud UMC, Nijmegen and Massachusetts General Hospital, in the first 6 months of 2018. Inclusion criteria for the radiographs were: patients aged between 18 and 90 years old; fracture classification AO Types A and C fractures; fractures with a dorsal angulation of the articular surface on the lateral radiograph close to threshold for acceptable alignment (between 0° and 25°), and good-quality radiographs (as measured by EB according to standardized methods) (Medoff 2005). Radiographs were classified by EB and checked by DR. Because we wanted to study the full spectrum of dorsal angulation, we included 23 radiographs (25%) with dorsal angulation between 0° and 5°, 48 radiographs (50%) between 6° and 15° dorsal angulation, and 24 radiographs (25%) between 16° and 25° of dorsal angulation. For each fracture a posteroanterior and lateral radiograph was presented. Radiographs at both institutions were taken according to similar positioning guidelines.

When studying interobserver variability, the study’s power is determined by the number of observers and the number of images. After a certain number of raters, power no longer increases; power can then only increase by rating more images. To make sure every rater did not have to review 95 radiographs we divided our 95 radiographs into 4 sets of 23 or 24 radiographs. Every participant was then randomized to 1 of the 4 survey sets with 23 to 24 radiographs.

Members of the SOVG were randomized to 1 of the 4 surveys each with 23 or 24 fractures. All observers were asked to indicate whether they would advise a reduction of a fracture of the distal radius. Every set of radiographs was accompanied by patient age and gender. Information on the criteria for selecting the radiographs was not added.

80 surgeons completed the survey, 72 were men, and the majority resided in Europe (n = 17) and North America (n = 51) (Table 1). 62 Surgeons were hand and wrist surgeons. Most of the surgeons supervised trainees (Table 1).

**Table 1.** Surgeon and clinical characteristics, study A. Values are count unless otherwise specified

Surgeon characteristics (n = 80)	
Men	72
Continent of practice	
United States	51
Europe	17
Other	12
Years in practice	
0–5	28
6–10	17
11–20	25
21–30	10
Supervising trainees	64
Main specialty	
Hand and wrist	62
Shoulder and elbow	15
Other	3
Clinical characteristics (n = 95)	
Age ^a^	61 (14)
Men	18
AO classification	
23-A	39
23-B	0
23-C	56
Dorsal angulation ^a^	11 (6.6)
AP distance ^a^	21 (2.5)
Radial height ^a^	7.4 (2.5)
Ulnar-ward inclination ^a^	12 (4.7)
Ulnar variance ^a^	–0.1 (2.0)

**^a^**Mean (SD)

Using a sample size calculation for Fleiss kappa, we calculated that a minimum of 94 images would allow us to find a kappa of 0.60 with a 95% confidence interval of 0.10 (half width), alpha set at 0.05 and 20 raters, assuming a proportion of 0.50 positive ratings for the recommendation for reduction study. Because we divided the available radiographs into 4 sets, we would need 80 observers.

### Influence of expert-based criteria on recommendations for a reduction

For the study addressing the influence of expert-based criteria on recommendation for a reduction, we selected 20 consecutive radiographs between November 2017 and February 2018 treated in the Radboud UMC, Nijmegen. Inclusion criteria for the radiographs were: patients aged between 18 and 90 years old, fracture classification AO types A and C fractures, fractures with a dorsal angulation near the threshold of acceptable alignment (dorsal angulation of 5 to 15 degrees), and good-quality radiographs. Radiographs were measured and classified by (EB [researcher]) and checked by a hand surgeon (DR). We included 5 radiographs with dorsal angulation between 5° and 7.5°, 10 radiographs between 7.6° and 12.5°, and 5 radiographs between 12.6° and 15°. All radiographs included a posteroanterior and lateral view of the fractured distal radius.

The criteria for when to reduce a DRF were expert based as there are no validated criteria for indication of a reduction after a DRF. The expert-based criteria were chosen by a panel of three trauma surgeons specialized in the upper extremity, and are based on the AAOS criteria for adequacy of DRF alignment. The criteria are: dorsal tilt of more than 10°, ulnar positive variance of more than 3 mm, radial inclination of less than 15°, intra-articular displacement if the fracture is intra-articular.

To investigate the influence of the expert-based criteria, the raters were randomized into 2 groups. Both groups received a survey with 20 sets of radiographs. One group also received the expert-based criteria for reduction. All observers were asked to indicate whether they would advise reduction of a DRF. Every set of radiographs was accompanied by patient age and sex.

68 surgeons completed the survey, 63 were men, and the majority were resident in Europe (n = 13) and North America (n = 43) (Table 2). 53 surgeons were hand and wrist surgeons. Most of the surgeons undertook supervision of trainees (Table 2).

**Table 2.** Surgeon characteristics, study B. Values are count

	Total n = 68	Without guideline n = 38	With guideline n = 30	p-value
Men	63	35	28	1.0
Continent of practice				0.3
United States	43	21	22	
Europe	13	9	4	
Other	12	8	4	
Years in practice				0.5
0–5	17	10	7	
6–10	16	11	5	
11–20	25	11	14	
21–30	10	6	4	
Supervising trainees	56	30	26	0.5
Main specialty				0.6
Hand and wrist	53	28	25	
Shoulder and elbow	13	9	4	
Other	2	1	1	

Assuming we needed a similar number of observers for the other study (influence of expert-based criteria on recommendation for reduction), 20 sets of radiographs would allow us to determine kappa with a 95% confidence interval of 0.18 (half width).

### Statistics

Continuous variables are described with means and standard deviations and categorical variables with absolute numbers.

We used the Fleiss kappa to assess the reliability (i.e., interobserver agreement) of advising a reduction for the DRF. We regarded non-overlapping 95% confidence intervals as a significant difference. The 95% confidence intervals were determined by bootstrapping (number of resamples: 1,000). Kappa values were interpreted using the classification of categorical data by Landis and Koch: a value of 0.01 to 0.20 indicates slight agreement; 0.21 to 0.40 fair agreement; 0.41 to 0.60 moderate agreement; 0.61 to 0.80 substantial agreement; and 0.81 to 0.99 near perfect agreement.

To determine factors associated with the likelihood of reduction we divided the proportion of recommended reductions by the total number of recommendations for each radiograph. We created a multivariable linear regression model with the likelihood for reduction as the dependent variable and the radiographic factors and patient characteristics as the independent variables.

### Ethics, funding, and potential conflicts of interest

This study received approval from the Institutional Review Board of the University of Texas at Austin, 2017-11-0081. The authors received no financial support for the research, authorship, and/or publication of this article. All the authors declare no conflict of interest related to this study.

## Results

### Interobserver reliability for recommending a reduction

Recommendation for reduction had fair interobserver reliability (kappa 0.3, 95% CI 0.2–0.4). Surgeons characteristics, age and sex of the patient, and fracture characteristics had no influence on the interobserver reliability (Table 3).

**Table 3.** Interobserver agreement (95% confidence interval) on recommending reduction

Factor	Kappa (95% CI)
Surgeon variables	
Overall	0.31 (0.23–0.39)
Sex	
Female	0.49 (0.29–0.69)
Male	0.30 (0.22–0.37)
Continent of practice	
USA	0.28 (0.19–0.37)
Europe	0.37 (0.27–0.47)
Other	0.45 (0.29–0.62)
Years of practice:	
0–5	0.36 (0.25–0.48)
6–10	0.28 (0.16–0.40)
11–20	0.20 (0.12–0.27)
21–30	0.36 (0.18–0.54)
Supervising trainees	
Yes	0.30 (0.22–0.37)
No	0.45 (0.32–0.59)
Main specialty	
Hand and wrist	0.29 (0.21–0.37) ^a^
Shoulder and elbow	0.42 (0.28–0.56)
Other	0.76 (0.42–1.10) ^a^
Set	
Set 1	0.28 (0.20–0.39)
Set 2	0.32 (0.12–0.52)
Set 3	0.31 (0.20–0.42)
Set 4	0.24 (0.11–0.36)
Patient variables	
Sex	
Female	0.31 (0.22–0.41)
Male	0.27 (0.14–0.40)
Age	
18–60	0.32 (0.21–0.43)
> 60	0.28 (0.18–0.38)
AO Classification	
23-A	0.40 (0.26–0.53)
23-C	0.21 (0.15–0.28)
Dorsal angulation (°)	
0–5	0.14 (0.04–0.24)
6–15	0.22 (0.12–0.32)
16–25	0.12 (0.06–0.18)
AP distance (mm)	
16–21	0.38 (0.27–0.50)
22–29	0.21 (0.14–0.29)
Radial height (mm):	
1–7.4	0.25 (0.12–0.37)
7.5–13	0.32 (0.21–0.42)
Radial inclination (°)	
1–11.6	0.25 (0.14–0.37)
> 11.6–23.6	0.31 (0.23–0.39)
Ulnar variance (mm)	
–4.5 to –0.8	0.34 (0.23–0.45)
> –0.8–5.4	0.26 (0.18–0.34)

**^a^**Statistically significant difference.

### Factors associated with recommending reduction

Multivariable linear regression analyses indicated that each additional degree of dorsal angulation increased the chance of recommending a reduction by 3% (beta 0.03, CI 0.02–0.03, p-value < 0.001) (Table 4, see Supplementary data). Dorsal angulation explained 37% of the variation in the likelihood of recommending a reduction (semi-partial R^2^ 0.4).

### Influence of expert-based criteria

Expert-based criteria for reduction did not increase the interobserver reliability for recommending a reduction (no criteria kappa 0.4, CI 0.3–0.6 vs. criteria 0.5, CI 0.3–0.6) (Table 5, see Supplementary data).

## Discussion

Surgeon biases, habits, and preferences contribute to variations in care. In the face of limited evidence, attitudes and beliefs concerning the indications for treatment are important reasons for surgical variation. Reducing unwarranted practice variation could lead to a reduction in avoidable morbidity and unhelpful use of resources (Birkmeyer et al. 2013). Our study addressed the interobserver reliability of surgeons recommending a reduction of a DRF and the influence of reading expert-based criteria influenced recommendations. This study was not intended to determine a threshold for when to reduce a distal radius fracture.

We acknowledge some limitations for the study. 1st, only 68 surgeons completed the influence of expert-based criteria study, and it might have been underpowered. We were close to our estimate, and power analysis for reliability studies is imperfect. Therefore, we do not think this had much influence on the study. 2nd, members of the SOVG are more likely to work in an academic setting than the average surgeon, which could decrease generalizability. 3rd, the SOVG group does not measure intraobserver reliability because it is always greater than interobserver reliability. 4th, our use of dorsal angulation (rather than ulnar variance or other criteria) as selection criteria, might have influenced the regression analysis. A similar study with different radiographic parameters as selection criteria might have slightly different results. 5th, measurements of the radiographic parameters were performed by one researcher and checked by the senior surgeon. Measurements are somewhat imprecise, but it is unlikely that there was any systematic bias and the random variations probably had little influence on the statistics. 6th, we did not ask the observers whether they would advise surgery or not. There may be a subset for which surgical considerations would alter recommendations for reduction, but that subset is likely to be small. Finally, there are important differences between a survey and actual practice. For example, the radiographs were accompanied only by age and sex. In actual practice more factors are important to determine whether a fracture needs reduction, for example patient occupation and hand dominance. In our opinion the relative simplification would be expected to reduce variability.

The fair interobserver reliability on whether to reduce a DRF or not (kappa 0.31, CI 0.23–0.39) is consistent with prior studies demonstrating notable variation and limited reliability in surgeon recommendations. The study by Tosti et al. (2014) found that the interobserver agreement for recommending treatment of little finger metacarpal neck fractures was fair. Until we have a better evidence base on whether to reduce a fracture or not, it may be worthwhile to invest in tools such as decision aids to help patients weigh the advantages and disadvantages and participate in the decision.

The observation that each additional degree of dorsal angulation increased the chance of recommending a reduction by 3% is consistent with our observation that surgeons often use dorsal angulation as the most important feature used to decide on whether to recommend reduction of a fracture. This is consistent with studies suggesting that dorsal angulation is one form of deformity after fracture that affects function measured with patient reported outcome measures (PROMs) such as the Disability of the Shoulder and Hand (DASH) score (McQueen and Caspers 1988, Gliatis et al. 2000, Wilcke et al. 2007, Ali et al. 2018). In a prior study similar to ours, radiographic parameters accounted for about half of the variation in treatment recommendations (Neuhaus et al. 2015). Other studies also show that patient factors such as male sex and age and fewer comorbidities did not explain any more of the variation in the treatment recommendation than radiographic factors alone (Mackenney 2006, Kodama et al. 2013).

The observation that expert-based criteria did not influence the interobserver reliability for recommending a reduction is consistent with prior reliability studies. For instance, exposure of observers to a description of staging of wrist arthritis related to scaphoid nonunion did not improve reliability of staging (ten Berg et al. 2017). This study and the study of Christensen et al. (1981) proposed that the interpretation of radiographs with a particular pathology involves the learned concept for what is normal and not normal, meaning that surgeons see what they already know or believe. Using additional guidance or knowledge could be less effective than expected due to the influence of cognitive biases such as anchoring and familiarity (Christensen et al. 1981, ten Berg et al. 2017). For instance, one study found that personality features influence treatment recommendations; a higher pioneer score (associated with innovation and creativity) was associated with a higher rate of recommendation for surgery (Teunis et al. 2015). This surgeon-characteristic influence on recommendation for treatment could have influenced the potential additional value of the expert-based criteria and may also be an explanation for observer variation. Further research should be conducted to investigate whether the expert-based criteria and especially dorsal angulation could increase the reliability among young residents and eventually limit practice variation.

In conclusion, limited interobserver reliability contributes to practice variation. There was notable variation in recommendations for reduction that was not accounted for by surgeon or patient factors and was not diminished by exposure to expert criteria. Dorsal angulation was the main driver for recommending a reduction, reflecting the fact that surgeons may focus on a few, relatively simplistic factors in making recommendations. The ability to learn from practice variation is hindered by notable variability that is, to date, unaccounted for by measured factors. Future studies might address surgeon cognitive bias and heuristics and the best methods for nudging people (patients and surgeons) toward evidence and what matters most to patients. In addition, future studies should assess the importance of the effect of dorsal angulation on deciding when to reduce a DRF and the influence on patient-reported outcome after DRF. 

## Supplementary data

Tables 4 and 5 are available as supplementary data in the online version of this article, http://dx.doi.org/10.1080/17453674.2020.1846853
